# Tibial Plateau Fractures: Not an Elegy to Football Practice

**DOI:** 10.7759/cureus.92295

**Published:** 2025-09-14

**Authors:** Tiago B Cunha, António Neto, Nuno Pereira, Cristina M Baptista, Maria Inês V Cabral, Diogo Portugal

**Affiliations:** 1 Physical Medicine and Rehabilitation, Centro de Medicina de Reabilitação de Alcoitão, Cascais, PRT; 2 Medicine, Centro de Medicina de Reabilitação de Alcoitão, Cascais, PRT

**Keywords:** football, muscle strengthening, rehabilitation, return to play, schatzker classification, tibial plate fracture

## Abstract

Tibial plate fractures are rare in sports and often associated with high-impact trauma. The most usual classification used in tibial plate fractures is the Schatzker Classification, graded from I to VI, according to the level of comminution of the lesion. There is a scarcity of reports of athletes who have suffered this kind of fracture while playing football or futsal. We present a report of a 37-year-old athlete who suffered a Schatzker V fracture while playing futsal. The athlete had surgical intervention by means of open reduction internal fixation with a plate and screws and was enrolled in a rehabilitation program focusing on quadriceps strengthening and gait training. Nine months after the fracture, our patient resumed playing, an outcome that is quite rare in these complex lesions.

## Introduction

Sports-related bone fractures may result from direct trauma or from overuse (stress fractures), particularly when training loads exceed the athlete’s physiological capacity [1].

Among these, tibial plateau fractures, which involve the intra-articular region of the knee, are relatively rare in football and futsal. They typically result from high-energy direct trauma to the joint, a mechanism less frequent in these sports. Alternatively, sudden changes of direction may induce pivoting movements of the knee, leading to associated ligamentous, meniscal, and occasionally osseous injuries.

Despite their rarity, tibial plateau fractures often necessitate prolonged periods of sporting inactivity and, in some cases, permanent cessation of athletic practice, owing to their intra-articular involvement. Most studies reporting on these fractures in athletes focus on the proportion who eventually return to sport, with few addressing the timeframe required for the return to play [2,3].

## Case presentation

We present the case of a 37-year-old male athlete, previously healthy, with regular participation in amateur seven-a-side football and futsal. He had a background as a federated football player until the age of 18, corresponding to a Tegner Activity Scale (TAS) level of 7/10 [4]. He lacked formal sports insurance due to the recreational nature of his current practice.

In May 2022, while playing in an amateur league match, he sustained direct trauma to the anterolateral aspect of his right leg following contact with an opponent, resulting in a fall with impact on the right knee. He immediately developed severe pain and functional impairment, preventing weight-bearing, and was transported to a tertiary emergency department.

**Figure 1:**
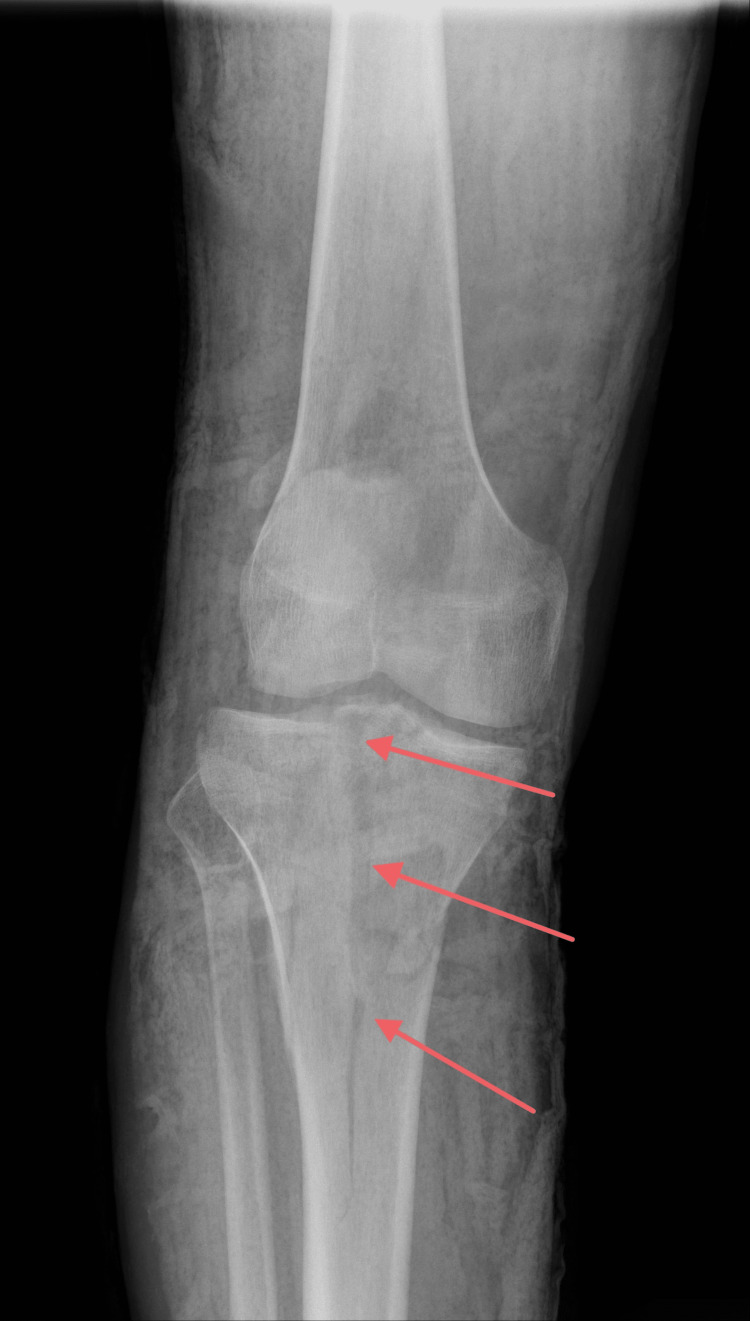
X-ray (AP view) showing a Schatzker type V fracture Red arrows point to the fracture line

**Figure 2:**
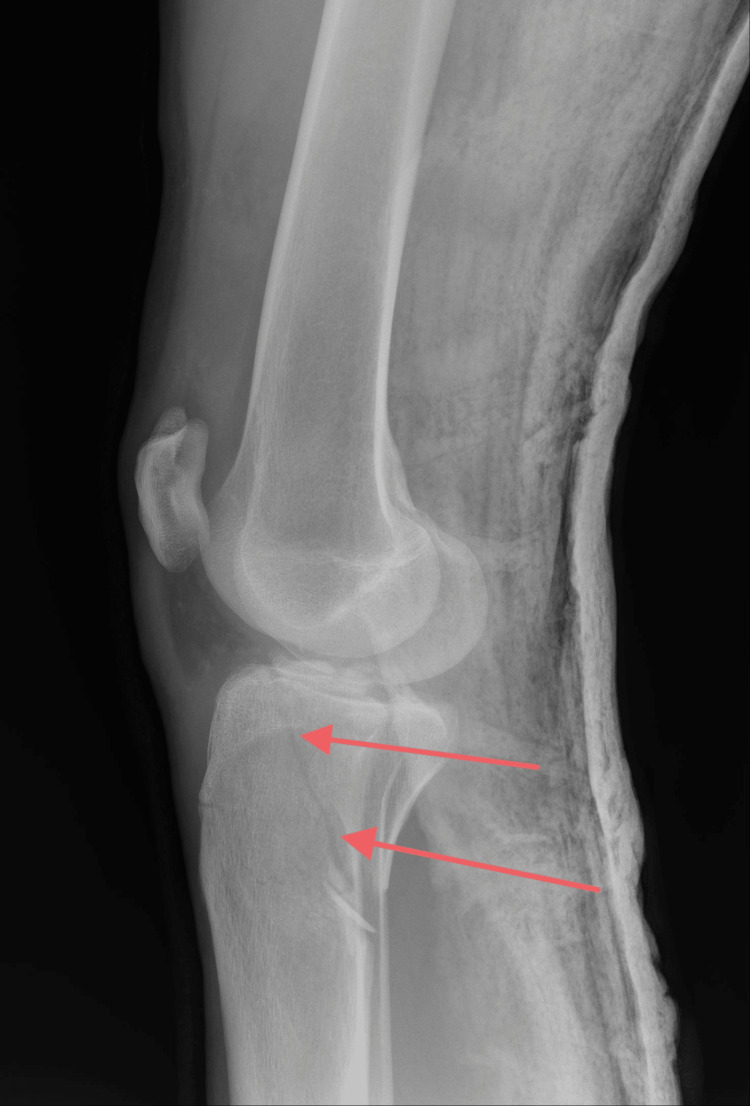
X-ray (Lateral view) showing a Schatzker type V fracture Red arrows point to the fracture line.

On examination, the patient was unable to ambulate and exhibited marked swelling of the right knee. Distal neurovascular status remained intact, with palpable peripheral pulses and preserved sensation. Initial anteroposterior and lateral radiographs, followed by computed tomography, revealed a Schatzker type V tibial plateau fracture [5], as shown in the pictures below (Figures 1, 2).

**Figure 3:**
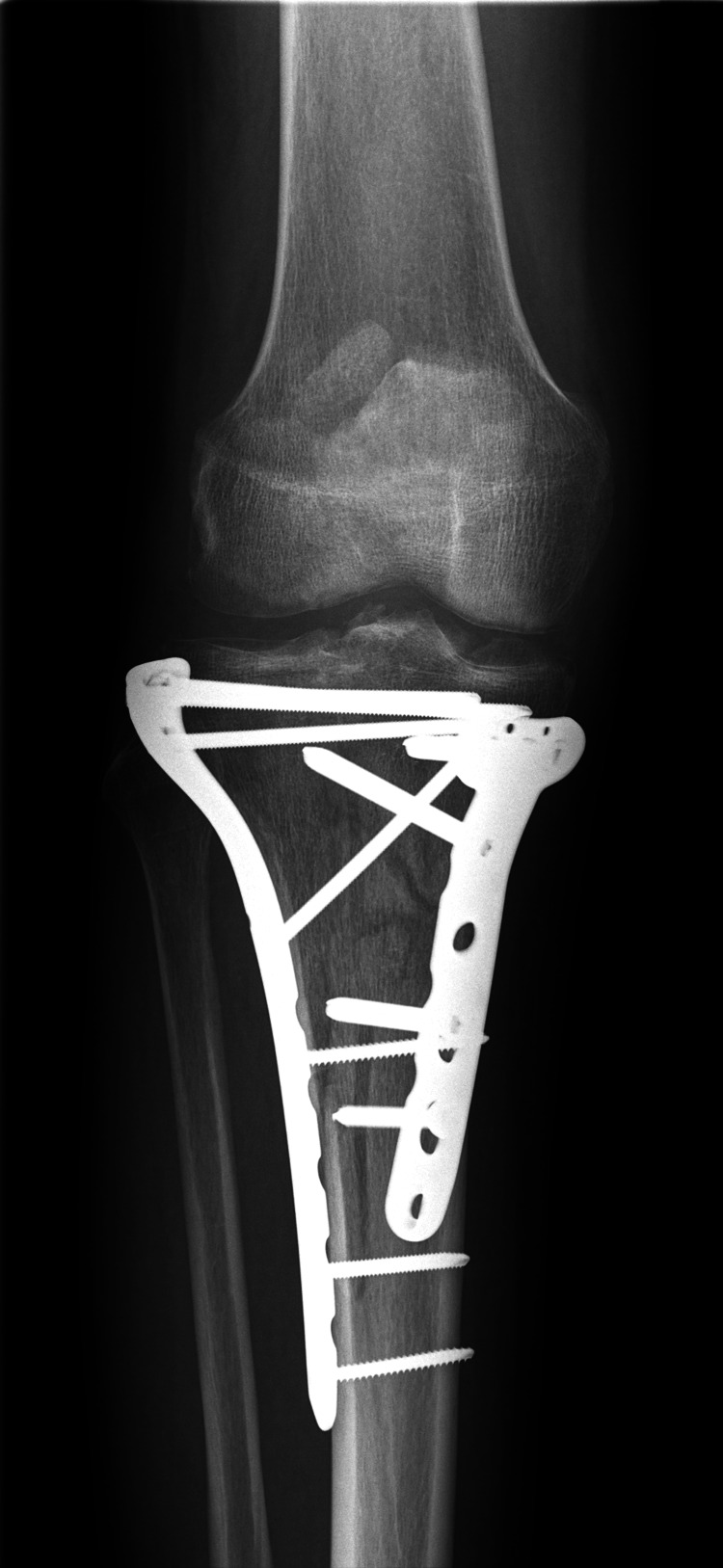
X-ray (AP view) showing the fracture after surgical intervention

**Figure 4:**
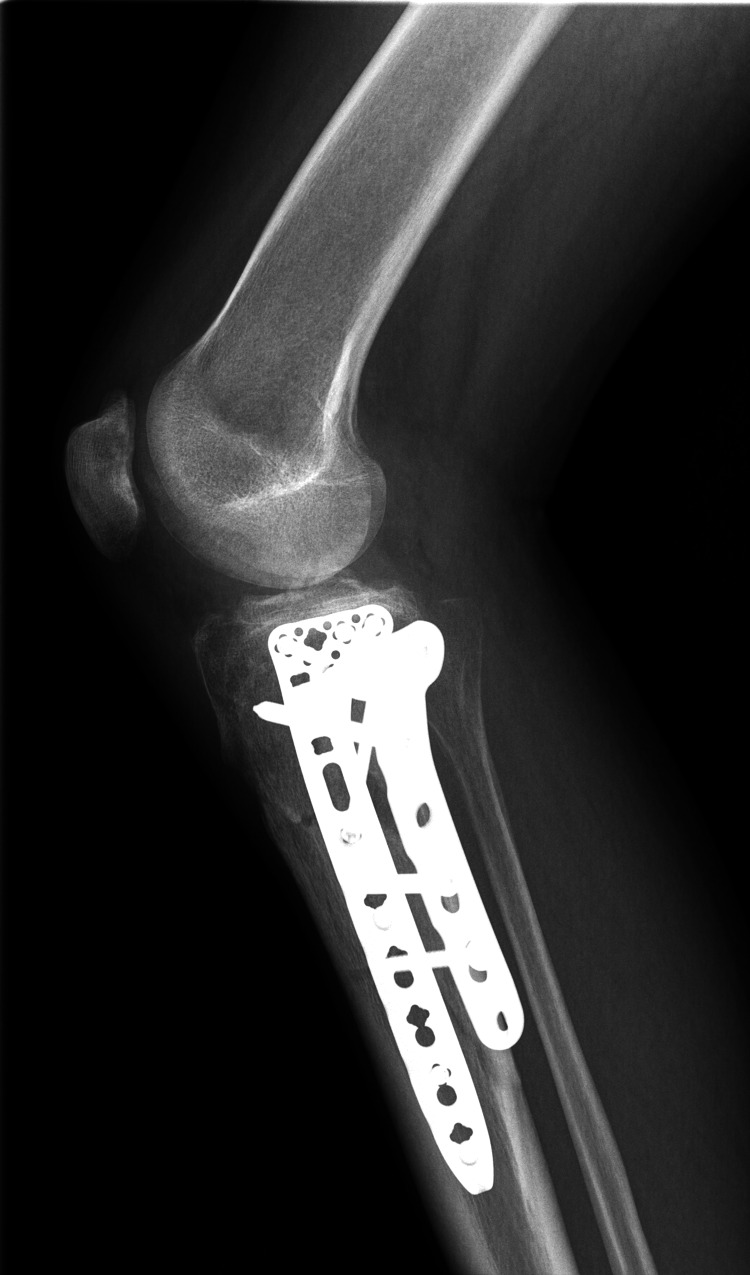
X-ray (Lateral view) showing the fracture after surgical intervention

The patient underwent open reduction and internal fixation (ORIF) with a plate and screws three days post-injury, as shown in the radiographs in Figures 3, 4.

Postoperatively, he commenced inpatient rehabilitation focusing on a progressive range of motion and non-selective quadriceps strengthening. Assisted ambulation with a walker began on postoperative day three, with strict non-weight-bearing on the affected limb.

The knee was immobilized in full extension using a Depuy brace for four weeks, after which joint mobilization exercises were initiated. At 12 weeks, partial weight-bearing commenced, with weekly increments of 25% of body weight until full loading was achieved. Outpatient rehabilitation continued thereafter, three times per week, emphasizing strength recovery and gait retraining.

At nine months post-injury, the athlete resumed football and futsal without functional limitations or pain. Analgesic requirements were minimal, limited to metamizole during the first postoperative week. Functional assessment yielded a Knee Outcome Survey-Activities of Daily Living Scale [6] score of 95.7% and a Sports Activities Scale [7] score of 85.5%, reflecting minor residual symptoms during sports participation compared to daily activities.

## Discussion

The Schatzker classification stratifies tibial plateau fractures from type I to VI, correlating increasing severity with higher-energy trauma and poorer prognosis [1]. The present case involved a type V fracture, characterized by bicondylar involvement, considered the second most severe category.

Contrary to expectations, associated soft tissue injuries do not consistently worsen functional outcomes. While meniscal injury is well documented in type IV fractures [8], there is limited evidence regarding concomitant soft tissue damage in Schatzker V and VI fractures. Some authors advocate for preoperative magnetic resonance imaging to better delineate associated ligamentous and meniscal injuries, potentially influencing surgical planning [9].

In football and futsal, most reported tibial plateau fractures are of lower severity, with surgical intervention rarely required. Athletes with type I or II injuries often undergo conservative treatment, including four to eight weeks of non-weight-bearing and immobilization, with return to competition typically no earlier than four months [2]. Protective equipment, such as shin guards, appears ineffective in preventing these injuries, as they predominantly shield the lower half of the leg [10,11].

## Conclusions

Despite being the most widely practiced sport worldwide, football has relatively few documented cases of tibial plateau fractures, and even fewer ones detailing return-to-play timelines. This case illustrates that even severe bicondylar fractures (Schatzker V/VI) do not necessarily preclude a successful return to sport. Favorable prognostic factors in this patient included younger age, good pre-injury physical condition, and the absence of neurovascular injury.

At nine months post-surgery, the athlete resumed his previous level of sports participation without pain. Nevertheless, accelerated progression toward post-traumatic osteoarthritis in the affected knee remains a concern, which may carry significant long-term financial implications given the absence of sports insurance.

## References

[1] Romani WA, Gieck JH, Perrin DH, Saliba EN, Kahler DM (2002). Mechanisms and management of stress fractures in physically active persons. J Athl Train.

[2] Robertson GA, Wong SJ, Wood AM (2017). Return to sport following tibial plateau fractures: a systematic review. World J Orthop.

[3] Kraml N, Haslhofer DJ, Winkler PW (2024). Tibial plateau fractures are associated with poor functional outcomes and a low conversion rate to total knee arthroplasty. Knee Surg Sports Traumatol Arthrosc.

[4] Tegner Y, Lysholm J (1985). Rating systems in the evaluation of knee ligament injuries. Clin Orthop Relat Res.

[5] Schatzker J, McBroom R, Bruce D (1979). The tibial plateau fracture. The Toronto experience 1968--1975. Clin Orthop Relat Res.

[6] Irrgang JJ, Snyder-Mackler L, Wainner RS, Fu FH, Harner CD (1998). Development of a patient-reported measure of function of the knee. J Bone Joint Surg Am.

[7] Borsa PA, Lephart SM, Irrgang JJ (1998). Comparison of performance-based and patient-reported measures of function in anterior-cruciate-ligament-deficient individuals. J Orthop Sports Phys Ther.

[8] Yan B, Sun J, Yin W (2021). The prevalence of soft tissue injuries in operative Schatzker type IV tibial plateau fractures. Arch Orthop Trauma Surg.

[9] Risitano S, Giustra F, Bosco F (2024). Tibial plateau fractures are associated with ligamentous and meniscal injuries. Preoperative evaluation of magnetic resonance imaging influences surgical treatment. Eur J Trauma Emerg Surg.

[10] Tatar Y, Ramazanoglu N, Camliguney AF, Saygi EK, Cotuk HB (2014). The effectiveness of shin guards used by football players. J Sports Sci Med.

[11] McKinley TO, Borrelli J Jr, D'Lima DD, Furman BD, Giannoudis PV (2010). Basic science of intra-articular fractures and posttraumatic osteoarthritis. J Orthop Trauma.

